# Comparative transcriptomes of three different skin sites for the Asiatic toad (*Bufo gargarizans*)

**DOI:** 10.7717/peerj.12993

**Published:** 2022-02-22

**Authors:** Yue Lan, Lewei He, Xue Dong, Ruixiang Tang, Wanyu Li, Jiao Wang, Lei Wang, Bisong Yue, Megan Price, Tao Guo, Zhenxin Fan

**Affiliations:** 1 Key Laboratory of Bioresources and Eco-Environment (Ministry of Education), College of Life Sciences, Sichuan University, Chengdu, Sichuan, China; 2 Department of Ambulatory surgery, West China Second University Hospital, Sichuan University, Chengdu, Sichuan, China; 3 Sichuan Key Laboratory of Conservation Biology on Endangered Wildlife, College of Life Sciences, Sichuan University, Chengdu, Sichuan, China; 4 Sichuan Engineering Research Center for Medicinal Animals, Xichang, Sichuan, China; 5 Department of Obstetrics and Gynecology, West China Second University Hospital, Sichuan University, kChengdu, Sichuan, China

**Keywords:** Bufo gargarizans, Amphibians, Transcriptomics, Differential expression gene, Genetic resource

## Abstract

Toads release toxic dry secretions from glands in their skin. Toxin possesses a wide range of biological effects, but little is known about its specific gene expression pattern and regulatory mechanisms. The Asiatic toad (*Bufo gargarizans*) is widely used to produce toxin. Here, we explored the gene expression of 30 tissue samples from three different skin sites (parotoid gland, dorsal skin, and abdomen skin) of *B. gargarizans*. After *de novo* assembly, 783,130 unigenes with an average length of 489 bp (N50 = 556 bp) were obtained. A total of 9,248 significant differentially expressed genes (DEGs) were detected. There were 8,819 DEGs between the parotoid gland and abdomen skin and 1,299 DEGs between the dorsal skin and abdomen skin, while only 1,283 DEGs were obtained between the parotoid gland and dorsal skin. Through enrichment analysis, it was found that the detected differential gene expressions corresponded to the different functions of different skin sites. Our key findings were the genetic expression of toxin secretion, the protection function of skin, and the related genes such as *HSD3B*, *Cyp2c*, and *CAT*, *LGALS9*. In conclusion, we provide useful transcript resources to study the gene expression and gene function of *B. gargarizans* and other amphibians. The detected DEGs between different sites of the skin provided better insights into the genetic mechanisms of toxin secretion and the protection function of skin for amphibians.

## Introduction

The Asiatic toad (*Bufo gargarizans*) belongs to the family of Bufonidae (Anura: Amphibia) and is widely distributed in East Asia. As a true toad, *B. gargarizans* has a pair of well-developed parotoid glands and numerous granular glands on the back (Lu et al., 2021). These glands secrete potent alkaloid toxins (collectively bufotoxins), which consist of structurally and functionally similar toxic compounds (*e.g.*, bufalin) (Hayes et al., 2009), to defend themselves against predators, parasites, and pathogens (Mina et al., 2015). In addition, the toxin derived from toads and the toxin possesses a wide range of biological effects, having long been used for the treatment of heart failure, tumors, sores, and pains in clinical settings in traditional Chinese medicine (Wu, Gao & Wang, 2010; Ji et al., 2014; State Pharmacopoeia Commission of the PRC, 2020).

In this study, we explored the question of what physiological differences exist in different skin sites in order to deal with the toxin presence. Because Amphibians played a very important role in the process of animal evolution and lived in a special ecological environment, they formed their own defense system (Zhou et al., 2021). The *B. gargarizans* could produce potent toxins to serve as weapons to fight their predators and to protect themselves (Ujvari et al., 2015). Physiological and biochemical analyses have identified the toxin contains chemical compounds classified into four categories: biogenic amines, bufadienolides, alkaloids, and peptides and proteins (Abdel-Rahman, Ahmed & Nabil, 2010; Mariano et al., 2018; Mariano et al., 2019). Some compounds belonged to the bufadienolides were considered the main active constituents of the toxin, such as bufalin, cinobufagin, arenobufagin, and resibufogenin (Li et al., 2021). Indole alkaloids were considered another type of active constituents of the toxin. Compared with the bufadienolides, there were relatively few reports on antitumor activity of Indole alkaloids (Li et al., 2021). One of the major active constituents of the toxin is bufalin (Wang et al., 2015; Liu et al., 2016). Although previous analyses have identified main compounds (Wu et al., 2020), the specific gene expression pattern and regulatory mechanisms of toxin and its components remain unclear.

RNA-seq is a powerful approach to analysis transcriptomes, which could generate global view of the transcriptional landscapes and correlate protein abundance with mRNA content (Wang, Gerstein & Snyder, 2009; Zhou et al., 2020; Wang et al., 2021). The transcriptomes of some tissues, such as liver, brain, heart, muscle and testicle from *B. gargarizans*, were sequenced (Yang, Qi & Fu, 2016; Yang et al., 2017; Jin et al., 2018), the gene expression profiles of skin were still missing. Yang et al. found the gene expression variations and genetic signals of high-altitude adaptation in *B. gargarizans* (Yang, Qi & Fu, 2016; Yang et al., 2017; Jin et al., 2018). Jin et al. (2018) sequenced liver transcriptomes of active and torpid B.gargarizans and gained insights into changes in gene expression patterns in hibernating female and male toads. Besides, although Lu et al. (2021) presented a high-quality genome assembly for *B. gargarizans*, the gene annotation was unavailable. Therefore, we applied transcriptomic methods to analyze the differentially expressed genes (DEGs) in different skin sites of *B. gargarizans* to explore the differential gene expressions. Transcriptome sequencing was performed on 30 samples from the parotoid gland, dorsal skin, and abdomen skin of ten individuals using Illumina paired-end sequencing technology. Our main purposes were to: (1) complete the assembly of the transcriptome of *B. gargarizans*; (2) annotate the skin transcriptome of *B. gargarizans*; (3) calculate and analyze gene expression; (4) screen out the DEGs among the three skin sites; (5) perform functional enrichment of DEGs to further clarify the gene expression profiles at different skin sites of *B. gargarizans* and (6) investigate the genetic mechanisms may related to self-protection, toxin secretion and production of anti-tumor components.

## Materials and Methods

### Experimental design and sample collection

Ten wild adult female Asiatic toads (*B. gargarizans*) were obtained from the wetlands by the lake in Chengdu, Sichuan Province, China in Mid-October 2018. All toads were captured on the same day and lived in similar environments. All animal experiments in this study were approved by the Ethics Committee of the College of Life Sciences, Sichuan University (number 20210309009). The ten toads were either anesthetized and euthanized. After euthanasia, 30 tissue samples were taken from three skin sites; parotoid gland, dorsal skin and abdomen skin. The 30 samples from the three skin sites were collected in cryotubes and immediately stored in liquid nitrogen for later RNA extraction and qPCR.

### Library preparation and RNA sequencing

Total RNA was isolated separately from the 30 samples using a TRIzol Kit (Invitrogen, Waltham, MA, USA) according to the manufacturer’s instructions. The construction of a cDNA library and transcriptome sequencing was conducted in Novogene (Beijing, China), using the PolyATract mRNA Isolation Systems Kit (Promega) and Illumina HiSeq 4000 according to the manufacturer’s instructions.

### Sequence preprocessing and transcripts assembly

**Figure 1:**
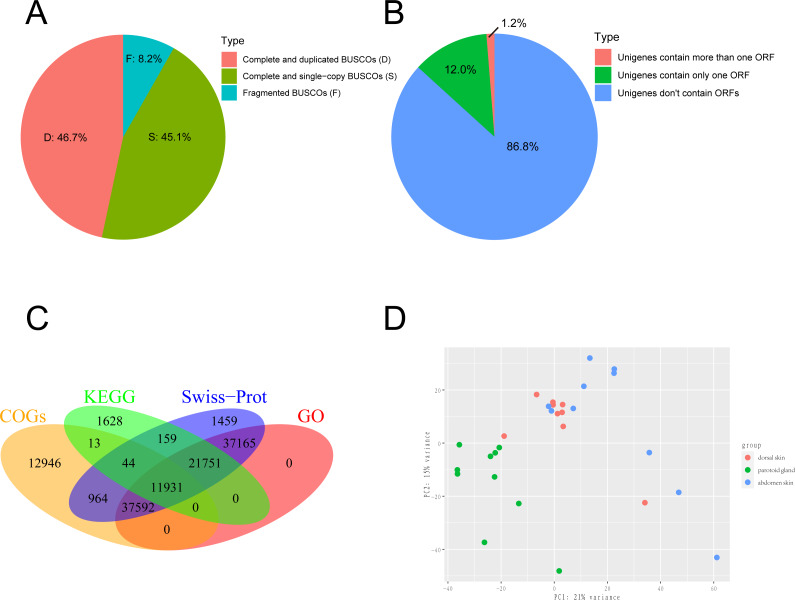
Sequencing and *de novo* assembly. (A) The results of BUSCO estimation. (B) Venn-diagram showing the classification of all identified open reading frames (ORFs). (C) Venn-diagram showing the overlap between results of unigenes aligned to Swiss-Prot, KEGG, COGs, and GO databases. (D) The principal component analysis (PCA) of 29 samples. This analysis used gene expression values, which normalized by Deseq2, and the differences between individuals are eliminated.

We obtained the 150-bp paired-end (PE) short reads after Illumina sequencing was completed. The adapters and low-quality reads in raw reads were removed by NGS QC Toolkit v2.3.3 (Patel, Mukesh & Liu, 2012) with stringent criteria (reads with more than 90% bases within ≥ 20 base quality (*Q*-value)) to obtain clean reads. The transcriptome was *de novo* assembled using the short-read assembly program Trinity (version 2.8.6) software with the option “-min_contig_length 300” (Grabherr et al., 2011; Haas et al., 2013). After assembly, we used TrinityStats.pl, which comes with Trinity, to counted the number, size and N50 of components and transcripts. The assembled transcripts were clustered by CD-HIT (version 4.8.1) (Fu et al., 2012) with the threshold of 95% similarity and 90% coverage of query sequences to obtain the non-redundant transcript sets as the unigenes. To evaluate the completeness, we employed Benchmarking Universal Single-Copy Orthologs (BUSCO v5.1.2; http://busco.ezlab.org/) to evaluate the gene set of the toad using Vertebrata data (Seppey, Manni & Zdobnov, 2019). The result of BUSCO was shown in Fig. 1A. The protein-coding regions prediction of assembled unigenes was undertaken using TransDecoder-v5.5.0 and the longest open reading frame (ORF) predicted for each contig sequence with a minimum of 100 amino acids long (*Haaset al.2013*). The result of ORF prediction was shown in Fig. 1B.

### Gene annotation

Unigenes from the *de novo* assembly were aligned into the UniProt database (Rolf et al., 2004) and Clusters of Orthologous Groups (COGs) (Koonin et al., 2004) using DIAMOND (version 2.0.7.145) (Huson & Buchfink, 2015). The unigenes were also aligned into the Kyoto Encyclopedia of Genes and Genomes (KEGG) (Gerlich & Neumann, 2000) database using KAAS (Yuki et al., 2007) using the single-directional best-hit (SBH) method to predict hypothetic function and the background gene set we selected were *Homo sapiens*, *Xenopus laevis*, *Xenopus tropicalis* and *Nanorana parkeri*. Unigenes aligned to the UniProt database were assigned to Gene Ontology (GO) (Ashburner et al., 2000) terms according to the idmapping_selected.tab file (last modified on 2021-02-10). The WEGO chart was generated by uploading the GO annotation results to http://wego.genomics.org.cn/ (Ye et al., 2018). The results of gene annotation were shown in Fig. 1C.

### Differentially expressed gene and gene enrichment analysis

**Figure 2:**
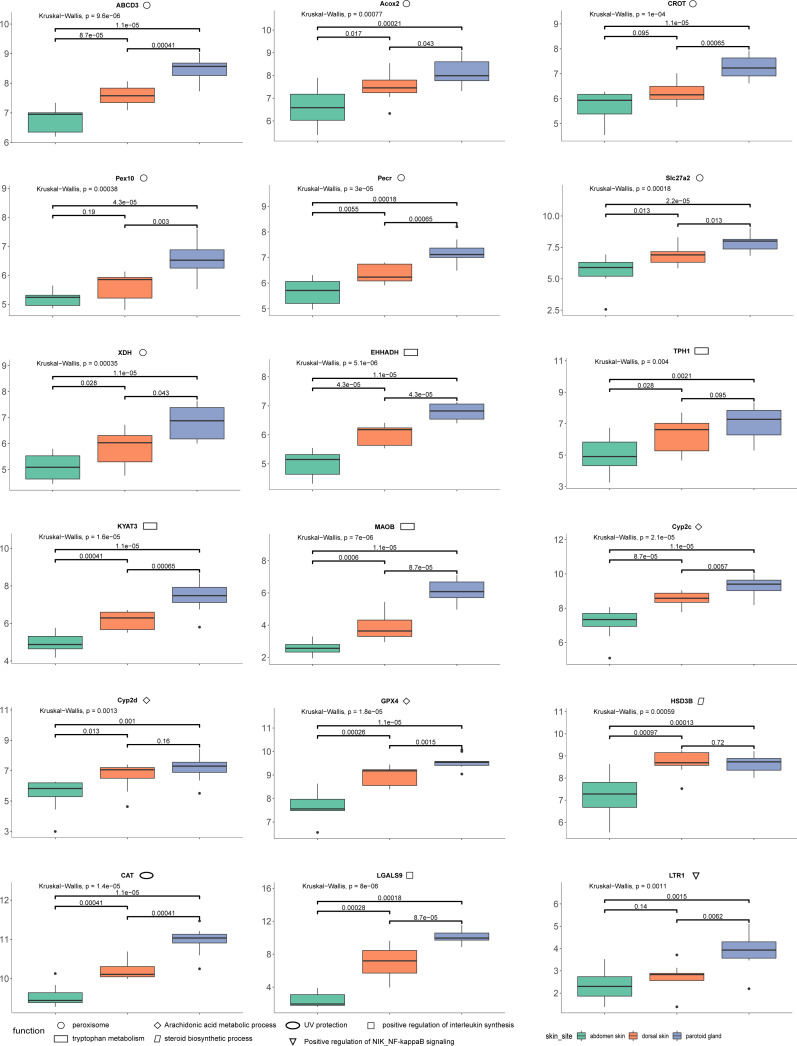
The results of differences in expression of 18 DEGs in three different skin parts. We calculate the 18 DEGs’ (*ABCD3*, *Acox2*, *CROT*, *Pex10*, *Pecr*, *Slc27a2*, *XDH*, *EHHADH*, *TPH1*, *KYAT3*, *MAOB*, *Cyp2c*, *Cyp2d*, *GPX4*, *HSD3B*, *CAT*, *LGALS9*, *LTR1*) expression level by the gene count matrix.

The quantification of these genes in each sample was performed using RNA-Seq by Expectation Maximization (RSEM) (Dewey & Li, 2011) after mapping to the assembled unigenes using Bowtie2 (version 2.4.2) (Langdon, 2015). We also mapped the samples to the reference genome of Asiatic toads from NCBI (GCA_014858855.1; Lu et al., 2021) using Bowtie2 (version 2.4.2) (Table S8). We eliminated the interference of low expression genes by removing genes from the gene count matrix obtained in the previous step that met all the following conditions: the number of samples with non-zero expression was less than ten. We then used R (version 3.6.2) package DESeq2 (Love, Huber & Anders, 2014) to identify the DEGs, with processed raw read counts as the input. We divided all samples into three groups according to the sampling site: dorsal skin, abdomen skin and parotoid gland. The read counts matrix and the samples’ information were constructed to a DESeqDataSet (dds) by the function, *DESeqDataSetFromMatrix*. After constructing dds, we performed a minimal pre-filtering to keep only rows that have at least two reads total. The standard differential expression analysis steps were wrapped into a single function, *DESeq*. Based on the normalized abundances of genes, principal component analysis (PCA) was performed using the function, *plotPCA* of DESeq2 (version 1.26.0) to visualize the correlation of the 30 RNA-seq samples (Fig. 1D). The Benjamini–Hochberg false discovery rate (FDR) was used to correct *P* values for multiple testing (FDR ≤ 0.05) and an absolute value of log 2 fold change (log2fc) was used to determine the significant differences in gene expression (log2fc ≥ 2). In this study, we focused on the DEGs that annotated using Swiss-Prot, GO and KEGG. GO and KEGG enrichment analyses were performed by R package clusterProfiler (version 3.14.3) (Yu et al., 2012) with GO and KEGG annotation results of all unigenes of the toad as the background gene set. After we completed the GO and KEGG enrichment of the differential genes, we selected the genes contained in the GO terms and KEGG pathways involved in the toxin secretion and self-protection of the toad. After we got the gene ids related to the toxin secretion and self-protection of the toad, we would get the list of UniProt Accession of these genes according to the Swiss-Prot annotation results (the dna_matches_fmt6.csv in the supplementary files). Finally, we would enter the list of UniProt Accession of these genes in bioDBnet (https://biodbnet-abcc.ncifcrf.gov/db/db2db.php) to get their gene symbols. The 18 genes we selected were shown in Fig. 2.

## Results

### Sampling, sequencing, and *de novo* assembly

We collected 30 tissue samples of three skin sites from ten wild adult female Asiatic toads (*B. gargarizans*) in Mid-October 2018. The three skin sites were the parotoid gland, dorsal skin, and abdomen skin. We then constructed 30 cDNA libraries and one of the dorsal skin samples was removed due to the unqualified cDNA library. Finally, a total of 813,227,650 raw reads were generated and 799,684,651 clean reads were obtained from 29 samples. Considering that the genome assembly of the Asiatic toads (*B.gargarizans*) lacked the corresoonding genome annotation file (.gtf file), we assembled the transcripts by *de novo* assembly. The clean reads were further assembled into 783,130 unigenes with an average length of 489.2 bp (N50 = 556 bp) after clustering and removing redundancy (Table 1). The average mapping rates of the samples that mapped to the reference genome and assembled unigenes were 62.41% and 88.58% respectively. We found that 45.1% of total complete and single-copy BUSCOs and 46.7% complete and duplicated BUSCOs were identified in this gene set, while 8.2% fragmented BUSCOs and nearly zero missing BUSCOs were identified in this gene set (Fig. 1A). A total of 117,553 ORFs were identified from 103,749 of the 783,130 assembled unigenes by TransDecoder, and 93,919 of the unigenes contained only one ORF (Fig. 1B).

**Table 1:** Summary of transcripts assembly.

total transcripts	783,130
max length	44,570
min length	201
total length	383106510.0
mean length	489.2
median length	327.0
Contig N10	2,627
Contig N50	556
Contig N90	241
GC content	46.22%

### Functional annotation of unigenes

We further annotated the function of the 783,130 assembled unigenes based on sequence similarity. Among these, 111,065 (14.00%), 35,526 (4.54%), 63,490 (8.11%) and 108,439 (13.80%) unigenes were aligned to Swiss-Prot, KEGG, COGs, and GO databases, respectively, and 11,931 unigenes were annotated across all databases (Fig. 1C). The GO annotation showed that the major subcategories were “Cell (GO:0005623)”, “Cell part (GO:0044464)”, “binding (GO:0005488)”, “catalytic activity (GO:0003824)”, “cellular process (GO:0009987)” and “metabolic process (GO:0008152)” (Fig. S1). COG assignments were performed to predict and classify the possible gene functions (Fig. S2). The hits from the COG prediction were classified into 26 functional categories, where the most enriched terms were translation, ribosomal structure and biogenesis (6,075 unigenes, J in Fig. S2), followed by amino acid transport and metabolism (5,860 unigenes, E in Fig. S2) and general function prediction only (5,116 unigenes, R in Fig. S2).

### Identification of DEGs and functional enrichments

We performed a PCA analysis on gene expression from the 29 samples with the normalized RNA-seq data and found that the three parts clearly clustered in the first Principal Component (PC1) and PC2, which explained 36% of the variance (Fig. 1D). After that, DEGs were analyzed with pairwise comparison of the three tissue types. A total of 9,248 significant DEGs were detected by DESeq2 (Table S1). DEGs with higher expression levels in one group, when compared to another, were denoted as “upregulated”, while those with lower expression levels were termed as “downregulated”. In general, 8,819 DEGs were identified between parotoid gland and abdomen skin, while only 1,283 DEGs were obtained between parotoid gland and dorsal skin. There were 1299 DEGs between dorsal skin and abdomen skin (Table S1).

We performed GO category and KEGG pathway enrichment analyses to gain insight into the biological roles of DEGs. First, GO and KEGG enrichment analyses were performed with 8819 DEGs between the parotoid gland and abdomen skin. Arachidonic acid metabolic process (GO:0019369), UV protection (GO:0009650), and catalase activity (GO:0004096) were mainly enriched by the upregulated DEGs in the parotoid gland (Table S2), whereas the downregulated DEGs in the parotoid gland were significantly enriched in extracellular matrix organization (GO:0030198), regulation of inflammatory response (GO:0050727), and calcium-dependent cysteine-type endopeptidase activity (GO:0004198) (Table S3). For KEGG enrichment, the upregulated DEGs in the parotoid gland were significantly enriched in peroxisome (ko04146), tryptophan metabolism (ko00380), and FoxO signaling pathway (ko04068) (Table S2), whereas the downregulated DEGs in the parotoid gland were significantly enriched in Calcium signaling pathway (ko04020) (Table S3).

Next, we performed GO and KEGG enrichment with the 1,299 DEGs found between dorsal skin and abdomen skin. The upregulated DEGs of dorsal skin were mainly enriched in steroid biosynthetic process (GO:0006694), arachidonic acid 14,15-epoxygenase activity (GO:0008404), and defense response to gram-negative bacterium (GO:0050829) (Table S4), while the downregulated DEGs of dorsal skin were enriched in melanin biosynthetic process (GO:0042438), embryonic forelimb morphogenesis (GO:0035515) and N-acetylmuramoyl-L-alanine amidase activity (GO:0008745) (Table S5). Glutathione metabolism (ko00480), Glutathione metabolism (ko00480), and Amphetamine addiction (ko00140) were significantly enriched by the upregulated DEGs of dorsal skin in the KEGG enrichment analysis (Table S4), while Neuroactive ligand–receptor interaction (ko04080), and Adrenergic signaling in cardiomyocytes (ko04261) were significantly enriched by the downregulated DEGs of dorsal skin in the KEGG enrichment analysis (Table S5).

Finally, we investigated the 1,283 DEGs identified between the parotoid gland and dorsal skin. The upregulated DEGs in the parotoid gland included enrichment in p38MAPK cascade (GO:0038066), positive regulation of CD4-positive, CD25-positive, alpha-beta regulatory T cell differentiation involved in immune response (GO:0032834), kynurenine metabolic process (GO:0070189), and cysteine-S-conjugate beta-lyase activity (GO:0047804) (Table S6). Whereas the downregulated DEGs in the parotoid gland were mainly enriched in xenobiotic metabolic process (GO:0006805) and calcium-dependent cysteine-type endopeptidase activity (GO:0004198) (Table S7). Glycosaminoglycan biosynthesis - chondroitin sulfate/dermatan sulfate (ko00532), and Amphetamine addiction (ko05031) were significantly enriched by the upregulated DEGs of the parotoid gland in KEGG enrichment (Table S6), while Nitrogen metabolism (ko00910) and Necroptosis (ko04217) were significantly enriched by the downregulated DEGs of the parotoid gland in KEGG enrichment (Table S7).

Combining our enrichment results and annotation results, we discovered 18 DEGs (ABCD3 (*p*-values: dorsal skin *vs* parotoid gland: 0.00041; dorsal skin *vs* abdomen skin: 0.000087; parotoid gland *vs* abdomen skin: 0.000011), Acox2 (*p*-values: dorsal skin *vs* parotoid gland: 0.043; dorsal skin *vs* abdomen skin: 0.017; parotoid gland *vs* abdomen skin: 0.00021), CAT (*p*-values: dorsal skin *vs* parotoid gland: 0.00041; dorsal skin *vs* abdomen skin: 0.00041; parotoid gland *vs* abdomen skin: 0.000011), CROT (*p*-values: dorsal skin *vs* parotoid gland: 0.00065; dorsal skin *vs* abdomen skin: 0.095; parotoid gland *vs* abdomen skin: 0.000011), Cyp2c (*p*-values: dorsal skin *vs* parotoid gland: 0.0057; dorsal skin *vs* abdomen skin: 0.000087; parotoid gland *vs* abdomen skin: 0.000011), Cyp2d (*p*-values: dorsal skin *vs* parotoid gland: 0.16; dorsal skin *vs* abdomen skin: 0.013; parotoid gland *vs* abdomen skin: 0.001), EHHADH (*p*-values: dorsal skin *vs* parotoid gland: 0.000043; dorsal skin *vs* abdomen skin: 0.000043; parotoid gland *vs* abdomen skin: 0.000011), GPX4 (*p*-values: dorsal skin *vs* parotoid gland: 0.0015; dorsal skin *vs* abdomen skin: 0.00026; parotoid gland *vs* abdomen skin: 0.000011), HSD3B (*p*-values: dorsal skin *vs* parotoid gland: 0.72; dorsal skin *vs* abdomen skin: 0.00097; parotoid gland *vs* abdomen skin: 0.00013), KYAT3 (*p*-values: dorsal skin *vs* parotoid gland: 0.00065; dorsal skin *vs* abdomen skin: 0.00041; parotoid gland *vs* abdomen skin: 0.000011), LGALS9 (*p*-values: dorsal skin *vs* parotoid gland: 0.000087; dorsal skin *vs* abdomen skin: 0.00028; parotoid gland *vs* abdomen skin: 0.00018), LTR1 (*p*-values: dorsal skin *vs* parotoid gland: 0.0062; dorsal skin *vs* abdomen skin: 0.14; parotoid gland *vs* abdomen skin: 0.0015), MAOB (*p*-values: dorsal skin *vs* parotoid gland: 0.000087; dorsal skin *vs* abdomen skin: 0.0006; parotoid gland *vs* abdomen skin: 0.000011), Pecr (*p*-values: dorsal skin *vs* parotoid gland: 0.00065; dorsal skin *vs* abdomen skin: 0.0055; parotoid gland *vs* abdomen skin: 0.00018), Pex10 (*p*-values: dorsal skin *vs* parotoid gland: 0.003; dorsal skin *vs* abdomen skin: 0.19; parotoid gland *vs* abdomen skin: 0.000043), Slc27a2 (*p*-values: dorsal skin *vs* parotoid gland: 0.013; dorsal skin *vs* abdomen skin: 0.013; parotoid gland *vs* abdomen skin: 0.000022), TPH1 (*p*-values: dorsal skin *vs* parotoid gland: 0.095; dorsal skin *vs* abdomen skin: 0.028; parotoid gland *vs* abdomen skin: 0.0021), XDH (*p*-values: dorsal skin *vs* parotoid gland: 0.043; dorsal skin *vs* abdomen skin: 0.028; parotoid gland *vs* abdomen skin: 0.000011)) that might relevant to toads’ self-protection and toxin secretion. We also calculate their expression levels in dorsal skin, parotoid glands and abdmen skin (Fig. 2).

## Discussion

To date, there have been only a few published complete genome sequences of amphibians mainly due to their large and complex genomes (Lu et al., 2021). Therefore, RNA-seq technology is a potentially useful tool to study their gene annotation, gene discovery, and gene expression (Geng et al., 2019). Recently, the complete genome sequence of the Asiatic toad was released (Lu et al., 2021), which provided a solid foundation to understand its genetic background, but the Asiatic toad genome still lacked the corresponding annotation files. In addition, there were some transcript resources of the Asiatic toads’ liver, brain, heart, muscle, testicles and oothecae (Yang, Qi & Fu, 2016; Yang et al., 2017; Jin et al., 2018), but the skin transcripts, an important tissue of the Asiatic toads, were still missing. In this study, we sequenced and analyzed the 29 skin transcriptomes from three sites on ten Asiatic toads. We constructed high-quality reference gene sets for the skin tissue and further analyzed the specific expression between different sites of the skin.

The toxin from dried secretions of the parotoid glands and skin glands of the Asiatic toad has been used in Traditional Chinese Medicine (TCM) for treating infection and inflammation for hundreds of years (Qi et al., 2014; Zhan et al., 2020). It has also been widely used as animal medicine in other Asian countries, such as Japan and Korea (The Korea Food and Drug Administration, 2016; Editorial board of Japan’s Ministry of Medicine, 2017). Both the parotoid gland and dorsal skin secrete toxin, but the white secretion from the parotoid gland produces the largest amount. Abdomen skin lacks the gland and thus cannot secrete toxin (Xia et al., 2019). We identified the largest number of DEGs (8,819) between the parotoid gland and abdomen skin, whereas there was only 1,283 DEGs between the parotoid gland and dorsal skin. The 1,283 DEGs between the parotoid gland and dorsal skin was also smaller than the 1,299 DEGs between dorsal skin and abdomen skin. The detected differential gene expressions were corresponding to the functions of different sites of the skin tissue. Therefore, the difference of gene expression profiles between the parotoid gland and abdomen skin was considerable, and the 8,819 DEGs may relate to the secretion of toxin.

We identified upregulated DEGs in the parotoid gland that were enriched in several important GO terms associated with toxin synthesis, such as Arachidonic acid metabolic process (GO:0019369). The DEGs enriched in Arachidonic acid metabolic process (GO:0019369) included *GPX4, Cyp2c* gene family (*CYP2C28*, *Cyp2c29*, *Cyp2c50, Cyp2c23, Cyp2c18, Cyp2c12, Cyp2c7, Cyp2c19, Cyp2c70* and *Cyp2c8*) (cytochrome P450 family 2 subfamily C) and *Cyp2d* gene family (*Cyp2d1*, *CYP2D6*, *Cyp2d26* and *CYP2D15*) (Fig. 2) (cytochrome P450 family 2 subfamily D). The protein encoded by *GPX4* belongs to the glutathione peroxidase family, members of which catalyze the reduction of hydrogen peroxide, organic hydroperoxides and lipid hydroperoxides, and thereby protect cells against oxidative damage (Huang & Wu, 2020). According to previous research, arachidonic acid was identified as a precursor of toxin (Bruzzone, Buschiazzo & Alonso, 2003). Bufalin, the primary active ingredient of toxin, is a cardiotonic steroid. Therefore, Arachidonic acid metabolic process (GO:0019369), steroid hydroxylase activity (GO:0008395), steroid delta-isomerase activity (GO:0004769) and epoxygenase P450 pathway (GO:0019373) may be associated with toxin secretion. Some of the DEGs are particularly important and need further study. For example, the DEG *HSD3B*, enriched in steroid biosynthetic process (GO:0006694), encoded an enzyme that catalyzes the oxidative conversion of delta-5-3-beta-hydroxysteroid precursors into delta-4-ketosteroids, which leads to the production of all classes of steroid hormones (Zein et al., 2020). The encoded protein also catalyzes the interconversion of 3-beta-hydroxy- and 3-keto-5-alpha-androstane steroids (Hearn et al., 2020). Furthermore, the *Cyp2c* and *Cyp2d* gene family that encode cytochrome P450 family 2 is involved in regulating the biosynthesis and metabolism of steroids (Zhang et al., 2019). The cytochrome P450 proteins are monooxygenases, which catalyze many reactions involved in the synthesis of cholesterol, steroids, and other lipids (Gómez-Tabales et al., 2020). The steroid is a water-soluble component in toxin (*Zhanget al.2019*). Steroids and bufalin isolated from toxin can induce apoptosis of human tumor cells by regulating the expression of the caspase gene family in cells (Gao et al., 2017).

In addition, several amino acid related terms or pathways were enriched with many upregulated parotoid gland DEGs, such as tryptophan metabolism (ko00380), which contained five DEGs (*CAT*, *MAOB*, *EHHADH*, *TPH1* and *KYAT3*) (Fig. 2). The *TPH1* encodes a member of the biopterin-dependent aromatic amino acid hydroxylase family (Choi et al., 2018). The encoded protein is one of two tryptophan hydroxylase enzymes that catalyze the first and rate the limiting step in the biosynthesis of the hormone and neurotransmitter, serotonin (Li & He, 2006). The encoded protein is involved in the development of hypoxia-induced elevations in pulmonary pressures and pulmonary vascular remodeling, and has also been implicated as a regulator of immune tolerance (Flamar et al., 2020). The *KYAT3* encodes an aminotransferase that transaminates kynurenine to form kynurenic acid, which is a metabolite of tryptophan (Matysik-Woźniak et al., 2017). The peroxisome (ko04146) was also enriched with many DEGs, such as *CAT*, *Slc27a2*, *EHHADH*, *Pex10*, *Pecr*, *CROT*, *Acox2*, *XDH* and *ABCD3* (Fig. 2). The protein encoded by *Slc27a2* is an isozyme of long-chain fatty-acid-coenzyme A ligase family (Tharp et al., 2016). Although differing in substrate specificity, subcellular localization, and tissue distribution, all isozymes of this family convert free long-chain fatty acids into fatty acyl-CoA esters, and thereby play a key role in lipid biosynthesis and fatty acid degradation (Veglia et al., 2019). The *CROT* encodes a member of the carnitine/choline acetyltransferase family. The encoded protein converts 4,8-dimethylnonanoyl-CoA to its corresponding carnitine ester. This transesterification occurs in the peroxisome and is necessary for transport of medium- and long- chain acyl- CoA molecules out of the peroxisome to the cytosol and mitochondria. The protein thus plays a role in lipid metabolism and fatty acid beta-oxidation (Okui et al., 2021). The product of *Acox2* belongs to the acyl-CoA oxidase family. It encodes the branched-chain acyl-CoA oxidase that is involved in the degradation of long branched fatty acids and bile acid intermediates in peroxisomes (Valentino et al., 2017).

According to previous studies, bufalin can enhance the level of interleukin in mouse lymphocytes with T lymphocyte-dependent immune response (Cho et al., 2018). Modern pharmacological studies show that bufalin has antitumor effects in various cancers, such as leukemia as well as lung, gastric, breast, prostate, ovarian and bladder cancers (*Liuet al.2016*). *LGALS9* is involved in the positive regulation of interleukin synthesis, so it can be inferred that *LGALS9* is a key gene involved in regulating the secretion of toxin (Qi et al., 2011).

In conjunction with the genetic expression of toxin secretion, we also explored the protective function from other environmental stressors of the skin. The skin is an important tissue to protect the Asiatic toad, which is the first line of defense against bacteria and other external stimuli (Gabriel & Costa, 2021). UV protection (GO:0009650) was enriched by the upregulated genes in the parotoid gland. The expression levels of *CAT* in the posterior ear glands and back skin were higher than in the abdominal skin (Fig. 2). The *CAT* gene is involved in the protection of the Chinese toad from ultraviolet rays (Muramatsu et al., 2005). This gene encodes catalase, a key antioxidant enzyme in the body’s defense against oxidative stress (Yenkoyan, Harutyunyan & Harutyunyan, 2018). The high expression level of these genes could play positive roles in the protection of the Chinese toad from ultraviolet rays. In addition, other genes that might relate to the survival of the Chinese toad were also observed as DEGs. For example, defense response to Gram-negative bacterium (GO:0050829), defense response to Gram-positive bacterium (GO:0050830), and cellular response to virus (GO:0098586) were enriched by the upregulated DEGs in dorsal skin and downregulated in abdomen skin. Positive regulation of NIK/NF-kappaB signaling (GO:1901224) was enriched by the upregulated DEGs in the dorsal skin. A family of Toll-like receptors (TLRs) acts as primary sensors that detect a wide variety of microbial components and elicit innate immune responses. All TLR signaling pathways culminate in activation of the transcription factor nuclear factor-kappaB (NF-κB), which controls the expression of an array of inflammatory cytokine genes (Kawai & Akira, 2007). The protein encoded by *LTR1* is a member of the Toll-like receptor (TLR) family which plays a fundamental role in pathogen recognition and activation of innate immunity. *LTR1* is ubiquitously expressed, and at higher levels than other TLR genes. They recognize pathogen-associated molecular patterns (PAMPs) that are expressed on infectious agents, and mediate the production of cytokines necessary for the development of effective immunity (Lanki et al., 2019).

In conclusion, we assembled and annotated a comprehensive gene set of the three different skin sites for *B. gargarizans*. This gene set could provide useful transcript resources to study the gene expression and gene function of *B. gargarizans* and other amphibians. The detected DEGs between different sites of the skin could provide better insights into the genetic mechanisms of toxin secretion and the protection function of the skin.

## Supplemental Information

10.7717/peerj.12993/supp-1 Supplemental Information 1 Functional classification of all the unigenes of the *B. gargarizans* based on Web Gene Ontology Annotation Plot

10.7717/peerj.12993/supp-2 Supplemental Information 2 Bar chart showing the results of unigenes aligned to COGs

10.7717/peerj.12993/supp-3 Supplemental Information 3 Supplementary tables

10.7717/peerj.12993/supp-4 Supplemental Information 4 The sequences of the 783,130 assembled unigenes, part 1

10.7717/peerj.12993/supp-5 Supplemental Information 5 The sequences of the 783,130 assembled unigenes, part 2

10.7717/peerj.12993/supp-6 Supplemental Information 6 The sequences of the 783,130 assembled unigenes, part 3

10.7717/peerj.12993/supp-7 Supplemental Information 7 The sequences of the 783,130 assembled unigenes, part 4

10.7717/peerj.12993/supp-8 Supplemental Information 8 The sequences of the 783,130 assembled unigenes, part 5

10.7717/peerj.12993/supp-9 Supplemental Information 9 The sequences of the 783,130 assembled unigenes, part 6

10.7717/peerj.12993/supp-10 Supplemental Information 10 The scripts and command lines used in this study

10.7717/peerj.12993/supp-11 Supplemental Information 11 The Swiss-Prot results of the assembled unigenes

## Additional Information and Declarations

### Data Availability

The following information was supplied regarding data availability: The raw RNA-seq data are available at the China National GeneBank DataBase (CDGBdb): CNP0001870.
