# Supplementary figures and images for: Comparative transcriptomes of three different skin sites for the Asiatic toad (Bufo gargarizans)
Source: PeerJ. 2022 Feb 22;10:e12993. doi: 10.7717/peerj.12993 (PMC8877344; doi:10.7717/peerj.12993)

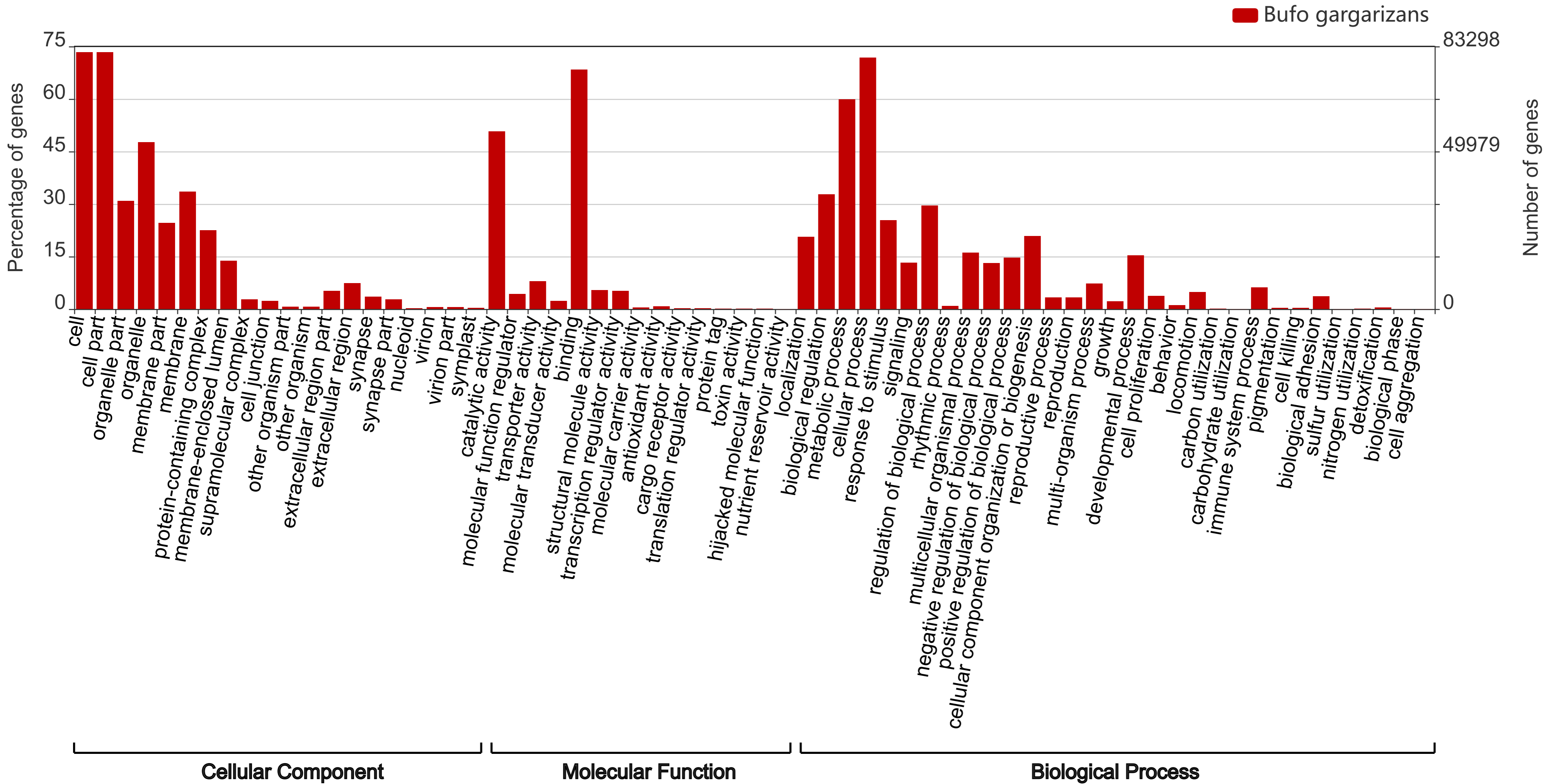

Supplement: Supplemental Information 1 [file peerj-10-12993-s001.pdf]

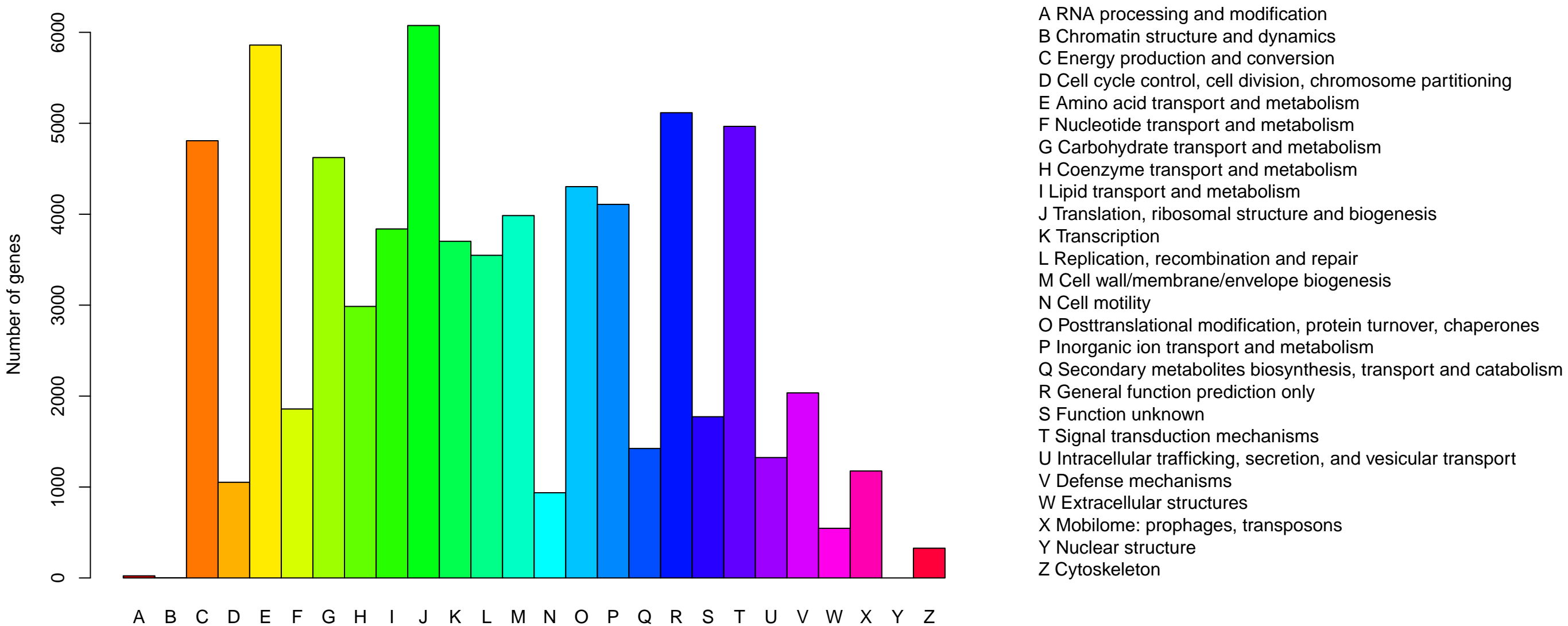

Supplement: Supplemental Information 2 [file peerj-10-12993-s002.pdf]
